# Imaging of chronic rhinosinusitis with nasal polyps in the era of biological therapies

**DOI:** 10.1097/ACI.0000000000000964

**Published:** 2024-02-12

**Authors:** Remo Poto, Corrado Pelaia, Antonio di Salvatore, Hesham Saleh, Guy W. Scadding, Gilda Varricchi

**Affiliations:** a Department of Translational Medical Sciences; b Center for Basic and Clinical Immunology Research (CISI), University of Naples Federico II; c World Allergy Organization (WAO) Center of Excellence, Naples; d Department of Health Sciences, University “Magna Graecia” of Catanzaro, Catanzaro, Italy; e Charing Cross Hospital, Imperial College Healthcare NHS Foundation Trust; f Allergy and Clinical Immunology, National Heart and Lung Institute, Imperial College, London, UK; g Institute of Experimental Endocrinology and Oncology (IEOS), National Research Council (CNR), Naples, Italy

**Keywords:** biological therapies, computed tomography, chronic rhinosinusitis with nasal polyps, imaging, magnetic resonance, nasal polyps

## Abstract

**Purpose of review:**

Chronic rhinosinusitis (CRS) is a chronic inflammatory disorder of the sinonasal cavities classified into two major phenotypes: CRS with nasal polyps (CRSwNP) and without nasal polyps (CRSsNP). The diagnosis of CRS is based on clinical symptoms associated with imaging and/or nasal endoscopy findings of mucosal inflammation.

**Recent findings:**

Recently, novel biological therapies have emerged as therapeutic options for CRSwNP. Imaging is helpful in deciding whether surgery is likely to be beneficial and in guiding surgery. It can also help demonstrate a clinical response to medical therapy. However, specific guidelines concerning the role of imaging in CRwNP are lacking.

**Summary:**

This article provides a comprehensive and critical multidisciplinary review of the role of conventional radiology, computed tomography (CT), and magnetic resonance imaging (MRI) in the diagnosis and characterization of CRSwNP. Since the complete characterization of nasal polyps on CT or MR images is very challenging, we provide a critical review of the best imaging methods and essential reporting elements used to assess nasal polyps.

## INTRODUCTION

Chronic rhinosinusitis (CRS) is a chronic inflammatory disorder of the sinonasal cavities [1^▪^], affecting 5–12% of the population worldwide [2–5]. Symptoms include anterior or posterior rhinorrhea, nasal congestion, hyposmia, and/or facial pressure or pain lasting over 12 weeks. The diagnosis of CRS is based on clinical symptoms associated with imaging and/or nasal endoscopy findings of mucosal inflammation [1^▪^]. CRS can be classified into two major phenotypes: CRS with nasal polyps (CRSwNP) and CRS without nasal polyps (CRSsNP) [6].

CRSwNP, which accounts for approximately 18–20% of all CRS cases, is the most debilitating phenotype with an onset of 42 years [7]. CRSwNP is associated with higher levels of morbidity and asthma severity, being found in 10–30% and 70–90% of mild and severe asthmatics, respectively [5,8,9]. Overall, asthma affects 30–70% of CRSwNP patients [10^▪^,^11^,^12^,^13^]. Patients with CRSwNP and comorbid asthma have more severe disease, which is characterized by high rates of nasal polyp recurrence [14] and glucocorticoid dependence compared to patients with asthma alone [15].

CRSwNP and asthma share common pathophysiological mechanisms, of which type 2 inflammation is the most important [1^▪^]. However, growing evidence suggests multiple endotypes within CRSwNP [16]. Defects in the sinonasal epithelial cell barrier, increased exposure to pathogenic and colonizing microbiota, and dysregulation of the host immune system contribute to disease pathogenesis [17].

Nasal polyps are inflammatory lesions, typically bilateral, that originate from the ethmoid sinuses. Recently, novel biological therapies have emerged as therapeutic options for CRSwNP. These monoclonal antibodies (mAbs) directly inhibit various specific mediators of type 2 inflammation, including interleukin (IL)-4, IL-5, IL-13, immunoglobulin E (IgE) and thymic stromal lymphopoietin (TSLP) [18–20].

In this context, imaging findings are useful in deciding the best treatment options and monitoring the response to biological therapy in patients with CRSwNP. Several imaging methods have been used for the diagnosis and management of CRS. However, imaging findings do not always correlate with symptoms [21]. It is estimated that 3% to 40% of asymptomatic patients may have sinus abnormalities on computed tomography (CT) [21]. Although evidence-based recommendations on imaging modalities are not directly provided in current consensus papers, strong evidence of their role as diagnostic and prognostic tools still has to be extrapolated. By analyzing more related studies, the evidence-based role of the various imaging methods can therefore be described [22]. 

**Box 1:**
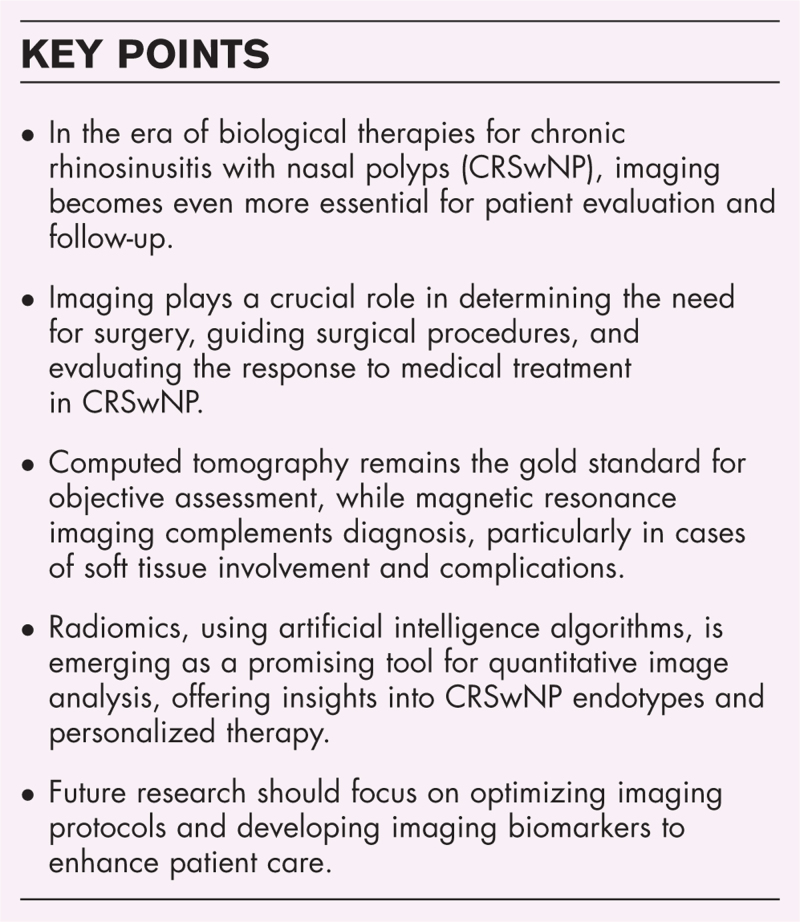
no caption available

## THE ROLE OF CONVENTIONAL RADIOGRAPHY IN CHRONIC RHINOSINUSITIS

Radiography was once the most commonly ordered study for evaluating the paranasal sinuses. Standard X-ray is still frequently used as an imaging procedure in patients with suspected CRS but even experienced radiologists may have difficulties in correct interpretation, particularly in judging whether mucoperiosteal thickening is present and whether the ethmoid and sphenoid sinuses are inflamed [23]. Although there is general agreement concerning the better accuracy of CT, standard X-ray has the advantages of availability, lower radiation dose and cost. The most common findings of CRS on conventional radiography are opacification of the maxillary sinus, followed by mucosal thickening and haziness of the maxillary sinus, and by air-fluid level [24]. Mucosal thickening is the most common diagnostic plain radiographic feature of CRS and the least predictive of this disease. Maxillary antra with plain radiographic interpretations of fluid levels, haziness and opacity have high specificities and positive predictive values of CRS. Compared with CT, radiography has shown a low sensitivity of 25–41% for all sinus groups except the maxillary sinuses with 80% sensitivity [23]. Burke and collaborators reported a plain film sensitivity of 70% and a specificity of 96–100% in the diagnosis of maxillary sinusitis [25]. However, conventional radiology is insufficient to evaluate endonasal drainages, anterior ethmoid cells, the frontal recess and two-thirds of the superior part of the nasal cavity. Due to the superpositioning of the structures and problems experienced for ideal positioning, this diagnostic tool is usually suboptimal, and extension of soft tissue mass and bone destruction are not clearly visualized [24]. Hence, conventional radiography should not be used as a single diagnostic tool in diagnosing and characterizing CRSwNP, especially in preoperative evaluation [24].

### The role of CT in chronic rhinosinusitis: advantages and limitations

CT is a reliable imaging method for detecting and localizing sinus opacifications. CT is frequently utilized as an objective diagnostic technique in CRS to differentiate between inflammatory and other sinus disorders and to determine the severity of CRS. CT provides extensive information on the anatomy of the paranasal sinuses and anatomical variants associated with CRS (e.g., nasal septal deviation, concha bullosa, Haller cells). CT scan is a mandatory diagnostic tool prior to endoscopic sinus surgery, and it is also employed as a “road map” during these procedures, providing key anatomical details for surgical planning.

CRS symptoms and endoscopic findings may overlap with those of rhinitis or neoplastic diseases. In such cases, a CT scan is necessary to support the suspected diagnosis. Unlike standard X-rays, CT provides objective information on the extent of sinus disease and is, therefore, the most common objective tool for staging the CRS.

To increase the diagnostic and prognostic values of CT, a variety of staging systems have been proposed. Among these, the Lund Mackay (LM) score [26] (Table 1), which assigns to each sinus cavity a score of 0 (no opacification), 1 (partial opacification), or 2 (total opacification) based on the extent of mucosal thickening within that sinus, plus a 0–2 score for the osteomeatal complex obstruction, appears to better quantify the severity of the disease in relation to other systems (e.g., the Kennedy, the Levine and May, the Friedman and the Harvard) [27–29].

**Table 1:** Lund Mackay score

Sinus	Right sinus	Left sinus
Frontal	0–2	0–2
Anterior ethmoids	0–2	0–2
Posterior ethmoids	0–2	0–2
Maxillary	0–2	0–2
Sphenoid	0–2	0–2
Ostiomeatal complex	0 or 2	0 or 2

For the sinuses: 0, no inflammation; 1, partial inflammation; 2, 100% inflammation. For the osteomeatal complex: 0, not occluded; 2, occluded. Maximum total score: 24.

The newly developed Amsterdam Classification on Completeness of Endoscopic Sinus Surgery (ACCESS) score is a CT-based scoring system grading the extent of surgery and predicting future surgery revisions [30^▪^]. Nevertheless, there are some crucial issues using CT scans, such as the radiation dose and the artifacts caused by implanted metals, which interfere with its accuracy. Finally, automated deep learning-based algorithms for quantitative sinus CT analysis are emerging [31].

### Prognostic value of CT in chronic rhinosinusitis

CT can capture the extent of disease and symptom severity, with a strong correlation between the extent of disease and polyp grade, both objective markers of disease severity [32]. A number of studies have failed to demonstrate significant correlations between CT scores and disease-specific health-related quality of life questionnaires, such as the SNOT-20, SNOT-22 and Chronic Sinusitis Survey (CSS) [33–36]. No significant association was determined between mucosal inflammation graded by histopathologic findings and clinical symptom scores. On the other hand, some studies reported a significant correlation between CT and symptom scores. Hanyu *et al.*[37^▪^] demonstrated a positive correlation between nasal symptoms and loss of smell, as well as an inverse correlation between pain and ocular symptoms, with objective radiological findings in patients with presurgical CRSwNP.

Sedaghat *et al.*[38] found that nasal symptoms, fatigue, and sleep disturbance of CRSwNP patients correlated with the severity of disease on CT scans, while headache and facial pain/pressure showed no correlation. However, nasal symptoms positively associated with CT score in the overall group of CRSwNP patients but not CRSsNP [39].

In clinical practice, Gregurić *et al.*[39] reported an association between the extent of sinus surgery, complications (recorded as revision surgery rate) and the severity of disease on cross-sectional imaging at sinus CT graded by LM score in CRSsNP. The association between complication rates and imaging at sinus CT is independent on the extent of surgery [39]. There is also evidence of a predictive value of the LM score in the prognosis of functional endoscopic sinus surgery (FESS). LM score of at least 13 is associated with better clinical outcomes in CRS patients undergoing FESS and thus can be used as a threshold to candidate patients for surgery. Nevertheless, similar analyses in patients with CRSwNP were not performed [40].

CT findings can help identify different CRS phenotypes [41^▪▪^]. Eosinophilic CRSwNP (eosCRSwNP) is associated with extensive sinus disease, severe symptom, and poor disease control compared with noneosCRSwNP [1^▪^,^42^]. Recent studies have analyzed the role of CT in differentiating eosinophilic and noneosinophilic CRSwNP [43]. The CT score and ratio for the ethmoid and maxillary sinus (E/M ratio) showed that the ethmoidal sinuses were more affected in eosinophilic CRSwNP. In contrast, noneosinophilic CRSwNP showed predominant maxillary involvement [43]. The E/M ratio also showed high accuracy as a predictor for CRSwNP recurrence [44^▪^].

Ishitoya and collaborators reported that CT scans of eosinophilic CRSwNP patients typically show ethmoid sinus-dominant opacification, with opacification of the olfactory cleft also a common feature [45]. The olfactory cleft score (Table 2) could be used as a supplementary predictor of glucocorticoid sensitivity in CRSwNP patients. There is evidence that olfactory cleft area opacification in sinus CT could be a prominent feature of glucocorticoid-sensitive CRSwNP and may help improve the prediction of glucocorticoid sensitivity in CRSwNP management [46].

**Table 2:** Grading system of the olfactory cleft opacification on computed tomography [47]

Grading	Anterior olfactory cleft	Posterior olfactory cleft
Grade 0	no opacification	No opacification
Grade 1	≤25% opacification	≤25% opacification
Grade 2	25–50% opacification	25–50% opacification
Grade 3	50–75% opacification	50–75% opacification
Grade 4	≥75% opacification	≥75% opacification

CT scores correlate with the histopathologic degree of inflammation in CRSwNP and CRSsNP [29,48,49]. Several studies have found a correlation between the severity of mucosal inflammation, as measured with the LM score, and osteitis. However, a widely accepted grading system for osteitis must be developed. CT scores also correlate significantly with type 2 cytokines [50].

### The role of CT in the follow-up of chronic rhinosinusitis with nasal polyps

**FIGURE 1:**
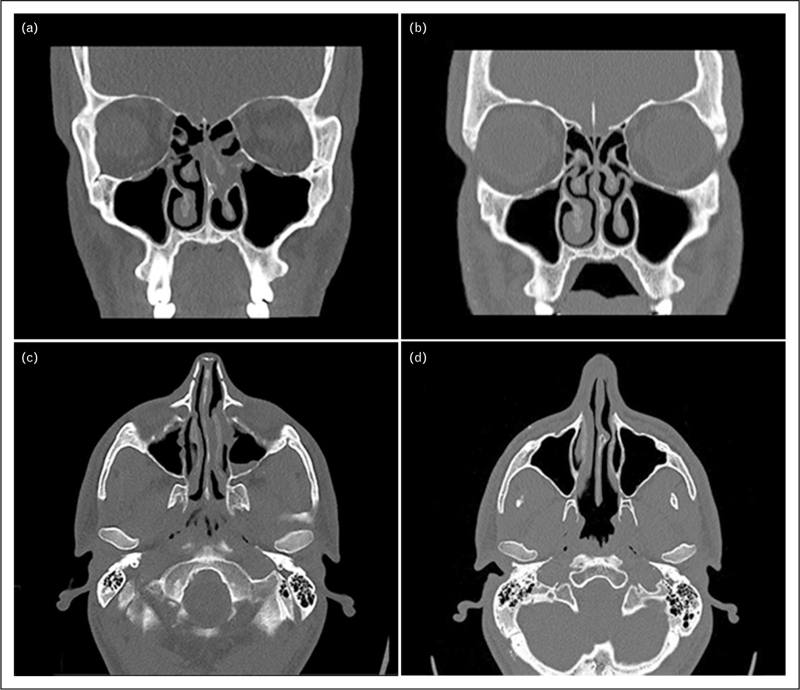
Coronal and axial CT scan performed before (a, c) and after (b, d) 16 weeks of dupilumab treatment (anti-ILRα) in a patient with CRSwNP. Images a and c show the presence of hypodense tissue with a polypoid appearance occluding the middle and upper portions of the left nasal fossa, along with signs of erosion of the ipsilateral middle turbinate. Images b and d show a significant reduction in the polypoid mass with normal pneumatization of the sinuses.

In the forthcoming era of biological treatments for uncontrolled CRS, imaging may be viewed as an outcome measure of objective improvement rate. CT has been employed as an outcome measure following medical treatment in several studies regarding oral and topical glucocorticoids, amphotericin, and capsaicin B [51]. To increase the sensitivity of the mucosal response, some studies measured mucosal thickness or nasal/sinus air volume before and after therapy. Gaveart *et al.*[52] showed an objective improvement after omalizumab (anti-IgE mAb) for CRSwNP evaluated using LM score before and 8–24 weeks after the treatment. Furthermore, Bachert *et al.*[53,54] showed a statistically significant reduction in the LM score in patients undergoing dupilumab treatment as early as 16 weeks. Figure 1 shows the rapid improvement of CRSwNP in a patient treated with dupilumab (anti-ILRα).

### Novel CT techniques

Multislice computed tomography (MSCT) is the imaging modality of choice in CRS with and without nasal polyps. Nonetheless, a higher cumulative radiation dose must be considered when repeated CT scans are performed. Radiation exposure during paranasal sinus CT examination is a major concern because the radiation-sensitive optic lens and thyroid gland are included in the scanning field [55].

Cone-beam computed tomography (CBCT) is a relatively new imaging technique for dentistry that, compared to conventional CT, delivers a lower radiation dose. CBCT scanning of the craniofacial region can be performed with an effective dose ranging from 30 to 80 microsieverts (Sv), whereas traditional MSCT imaging of the paranasal sinuses delivers approximately 860 Sv. The primary advantages of CBCT are lower cost and radiation dose, 3D reconstruction techniques and prospective applications in the temporal bone, skull base and sinus imaging. Compelling evidence suggests that CBCT may be helpful for intraoperative and postoperative bone structural evaluation, including intraoperative navigation; however, evidence regarding the clinical value of CBCT in diagnosing or staging CRS is limited [56,57].

## THE ROLE OF MRI IN CHRONIC RHINOSINUSITIS: ADVANTAGES AND LIMITATIONS

Magnetic resonance imaging (MRI) is a noninvasive imaging modality that has been increasingly utilized in the management of CRS. MRI can depict paranasal sinuses without radiation exposure, especially in cases involving children or when repeated examinations are needed. MRI can detect CRS, including key abnormalities such as mucosal swelling, mucopyoceles, nasal polyps, and medial maxillary sinus wall deformation [58]. The better differentiation of soft tissue by MRI allows for the determination of specific structural abnormalities of the paranasal sinuses that may otherwise not be differentiated by CT [59^▪^]. As mucosal swelling is one of the most prevalent signs of early sinonasal disease, MRI is well suited for the early detection of structural abnormalities in the paranasal sinuses. MRI may be useful in cases of diagnostic uncertainty after a CT scan, unilateral lesions, or when intracranial complications are suspected [59^▪^]. MRI can better distinguish solid masses from retained secretions [60].

MRI is the imaging modality of choice in cases with soft tissue involvement, orbital and intracranial complications, and to exclude or confirm cavernous sinus thrombosis [61,62].

MRI sinus imaging is better than CT in the differential diagnosis of sinusitis, polyps and malignancies, but it is commonly regarded to overestimate hypoplastic sinus disease compared to CT [22]. Therefore, MRI and CT can be considered as complementary techniques for imaging of the paranasal sinuses [22].

The relatively high cost of MRI examination and the presence of specific metal prostheses can limit its clinical application. MRI requires more time and is inferior to CT for characterizing bone structures. Although MRI occasionally requires sedation in young children, the ionizing radiation risk from CT scanning is of greater concern [63,64].

### Prognostic value of MRI in chronic rhinosinusitis

T2 value derived from T2 mapping has been proposed as a promising imaging biomarker for predicting eosCRSwNP [65^▪▪^]. T2 value of the nasal polyps was significantly higher in eosCRSwNP compared to noneosCRSwNP [65^▪▪^]. Moreover, T2 value was positively correlated with eosinophil count and negatively correlated with lymphocyte count in CRSwNP [65^▪▪^]. Indeed, Inflammatory cells can lead to increased edema in nasal polyps in patients with eosCRSwNP. By contrast, more fibrosis and less edema decrease T2 relaxation time in noneosCRSwNP tissues, resulting in a higher T2 value in eosCRSwNP than in noneosCRSwNP. Therefore, the T2 value may indicate eosinophil infiltration in CRSwNP tissues and be a noninvasive quantitative biomarker in predicting eosCRSwNP.

MRI has also been proposed as an effective imaging tool for evaluating the effects of glucocorticoid therapy in CRSwNP patients [66]. Correlations between the reduction of symptoms in VAS and the reduction of sinusitis have been reported with MRI [66]. A clinical trial (NCT03979716) is evaluating the brain activity differences in olfactory areas in CRSwNP patients before and after surgery using functional MRI to predict recovery of smell. MRI is also important in differentiating chronic invasive fungal rhinosinusitis from sinonasal squamous cell carcinoma [67].

### The role of MRI in the follow-up of chronic rhinosinusitis with nasal polyps

MRI can be a useful tool in the follow-up of patients with CRSwNP as it provides detailed visualization of the nasal and sinus tissues. In addition, MRI can be used for the follow-up of patients with CRSwNP to monitor treatment response to biological therapies. For example, it can help determine if the size of the polyps is decreasing or if there is improvement in the inflammation of the nasal and sinus tissues. It is important to note that MRI findings should be interpreted in conjunction with clinical assessment and patient history. Some patients with mild symptoms may not require MRI imaging, while others with more severe symptoms may benefit from regular follow-up MRIs to monitor the progression of the disease. Additionally, MRI findings must be interpreted cautiously, as some abnormalities detected on imaging may not necessarily correlate with symptoms or disease severity. Longitudinal paranasal sinus MRI is particularly useful in monitoring the progression of CRSwNP over time, especially in patients at high risk for disease recurrence or complications. By tracking changes in nasal and sinus tissue inflammation, MRI can help identify patients who may require more aggressive treatment or closer monitoring. However, it is crucial to acknowledge that MRI is currently associated with high costs and time-consuming procedures, which may limit its frequent utilization for monitoring CRS.

### Novel MRI techniques

In recent years, there have been significant advancements in MRI technology and techniques, including the development of multiparametric MRI (mpMRI). This novel imaging modality combines morphological and functional images, enabling a more comprehensive evaluation of the nasal and sinus tissues. MpMRI utilizes imaging sequences to generate high-resolution morphological images that depict the structure and anatomy of the nasal and sinus cavities. mpMRI also provides functional images that can provide information on tissue characteristics such as cellularity and vascularity. This information can be useful in identifying and characterizing CRSwNP. Furthermore, mpMRI has the potential to improve the accuracy of diagnosis and treatment planning. For instance, it can help differentiate between benign and malignant nasal tumors, guiding appropriate treatment decisions [68^▪^]. Additionally, mpMRI can help evaluate treatment response and monitor disease progression. However, more research is needed to fully evaluate the clinical utility of mpMRI and its potential role in the management of CRSwNP.

### Radiomics in chronic rhinosinusitis with nasal polyps

In the era of biological therapies, novel noninvasive methods are needed to identify endotypes of CRSwNP to facilitate personalized therapy. The latest developments in radiomics tools that use artificial intelligence algorithms are offering increasingly precise diagnoses. Radiomics has emerged as a promising tool for the quantitative analysis of medical images, offering new insights into the underlying pathophysiology of CRSwNP. Radiomics studies continue to improve prognosis and therapeutic response prediction, paving the way for imaging-based precision medicine. Radiomics involves extracting large amounts of quantitative data from medical images using advanced computational techniques, such as machine learning algorithms. These data can include a range of features, such as texture, shape, and intensity, which can be used to create predictive models and biomarkers for disease diagnosis, progression, and response to biological therapies.

In the context of CRSwNP, radiomics has the potential to provide new insights into the underlying pathophysiology of the disease. Radiomic analysis of CT scans can identify subtle changes in tissue texture and density, which may indicate inflammation or fibrosis. Radiomics features could help to identify eosinophilic vs. noneosinophilic endophenotypes and provide predictors of response to biological therapies. A recent study has investigated the potential of radiomics in diagnosing and managing CRSwNP [69^▪▪^], and a CT radiomics-based model has been developed to identify eosCRSwNP [69^▪▪^]. This radiomics-based method may provide novel insights in solving clinical concerns, such as guiding personalized treatment and predicting prognosis in patients with CRSwNP.

## CONCLUSION

In the era of biological therapies, imaging has become even more important for the evaluation and follow-up of patients with CRSwNP. Biologic agents such as mAbs targeting IL-4Ra have been shown to significantly reduce nasal polyp burden and improve symptoms in patients with CRSwNP. Imaging can help to assess treatment response and disease progression and may also aid in patient selection for biological therapies. Clinical suspicion, availability, cost and quality of information that obtained should guide the choice of the most appropriate imaging method.

Cone beam computed tomography (CBCT) is the preferred imaging method not only in CRSwNP but also in odontogenic sinusitis, noninvasive fungal infections, posttraumatic rhinorrhoea, benign tumors, whereas multidetector computed tomography (MDCT) is also useful for complicated acute sinusitis and invasive fungal infections.

MRI allows for better tissue characterization through T1, T2 sequences and fat signal suppression. Diffusion sequences (DWI/ADC), now commonly used in oncological imaging, allow the identification of highly cellular tissues and the acquisition of quantitative data. T2 value derived from T2 mapping of nasal polyps is a promising noninvasive quantitative parameter for identifying eosCRSwNP. The better differentiation of soft tissue by MRI allows for the determination of specific structural abnormalities of the paranasal sinuses that may otherwise not be differentiated by CT. MRI should also be preferred in the presence of spontaneous rhinoliquorrhea, cancer and invasive lesions. As CT is often used as an outcome measure after biological therapies (i.e., LM score), we envision that MRI could increase the sensitivity of mucosal response (e.g., mucosal thickness or nasal/sinus air volume before and after the treatment). Therefore, MRI could be useful not only for better tissue characterization of polyps but for the follow-up of CRSwNP patients undergoing biological therapies.

In conclusion, imaging plays a critical role in the management of CRS, particularly in patients with CRSwNP. CT scan and MRI are both valuable imaging modalities for the evaluation of CRS and may have complementary roles in specific clinical scenarios. With the emergence of biological therapies for the treatment of CRSwNP, imaging has become even more important for the evaluation and follow-up of these patients. Future research should focus on optimizing imaging protocols and developing imaging biomarkers to improve the diagnosis, management, and outcomes of patients with CRS.

## Acknowledgements

*The authors thank Dr Gjada Criscuolo for her excellent managerial assistance in preparing this manuscript, and the administrative staff (Dr Roberto Bifulco, Dr Anna Ferraro, and Dr Maria Cristina Fucci) without whom it would not be possible to work as a team.*

### Financial support and sponsorship

*This work was supported in part by grants from the CISI-Lab Project (University of Naples Federico II), TIMING Project and Campania Bioscience (Regione Campania).*

### Conflicts of interest

*There are no conflicts of interest.*

## REFERENCES AND RECOMMENDED READING

Papers of particular interest, published within the annual period of review, have been highlighted as:

▪ of special interest
▪▪ of outstanding interest
